# Weight loss in adolescents with down syndrome compared to adolescents with other intellectual disabilities enrolled in an 18-month randomized weight management trial

**DOI:** 10.3389/fped.2022.1022738

**Published:** 2022-11-02

**Authors:** Lauren T. Ptomey, Amy E. Bodde, Mary Hastert, Kameron B. Suire, Brian C. Helsel, Anna M. Gorczyca, Richard A. Washburn, Annie M. Rice, Joseph E. Donnelly

**Affiliations:** ^1^ Department of Internal Medicine, The University of Kansas Medical Center, Kansas, KS, United States; ^2^ Department of Dietetics and Nutrition, The University of Kansas Medical Center, Rainbow, KS, United States; ^3^ Department of Neurology, The University of Kansas Medical Center, Rainbow, KS, United States.

**Keywords:** down syndrome, diet, physical activity, remote delivery, body composition, weight loss, weight maintenance

## Abstract

**Background:**

There is limited information on the efficacy of weight management interventions in adolescents with Down Syndrome (DS)

**Objective:**

To compare weight change and intervention compliance between adolescents with DS compared to adolescents with non-DS related intellectual disabilities (ID) who were enrolled in an 18-month weight management trial.

**Methods:**

Participants were adolescents (13–21 years) with mild to moderate ID and overweight or obesity. Participants were randomized in a 1:1:1 allocation to one of 3 intervention arms for an 18-month weight management trial: face-to-face/conventional diet (FTF/CD), remote delivery/conventional diet (RD/CD), or remote delivery/enhanced Stop Light Diet (RD/eSLD). Anthropometrics were assessed at baseline 6, 12, and 18 months by staff blinded to the intervention, and self-monitoring data was collected across the 18-month study. As an unpowered, post-hoc, secondary analysis, two-sample *t*-tests were used to compare the weight change across 6,12, and 18 mos. and compliance across 18 mos. between adolescents with and without DS randomized to each intervention arm.

**Results:**

Adolescents with ID (*n* = 110) were randomized to one of three intervention arms: FTF/CD (*n* = 36, DS = 17, other ID = 19), RD/CD (*n* = 39, DS = 21, other ID = 18) or RD/eSLD (*n* = 35, DS = 15, other ID = 20). Body weight at 18 months was obtained from 82%, 76% and 73% of participants with DS and 84%, 83% and 75% of participants with other ID randomized to the FTF/CD, RD/CD, and RD/eSLD arms, respectively Weight change across 18 months was −0.2 ± 8.8 kg (−0.5%), −0.3 ± 5.3 kg (−0.7%), and −2.6 ± 5.0 kg (−4.0%) in adolescents with DS randomized to the FTF/CD, RD/CD and RD/eSLD arms, respectively. There were no significant differences in change in body weight or BMI across 18 months between adolescents with DS or those with other ID in any of the 3 intervention arms (all *p* > 0.05). Additionally, there were no significant differences in intervention compliance between adolescents with and without DS across 18 mos. (all *p *> 0.05).

**Conclusions:**

Adolescents with DS respond to a multi-component weight management intervention similar to those with others ID

## Introduction

Down syndrome (DS), a genetic condition caused by extra chromosome 21 material in all or some cells, is the most common chromosomal abnormality associated with intellectual disability (ID) (1) with an estimated prevalence of 12.7 per 10,000 among children age 0–4 years in the United States (2). The prevalence of overweight (BMI-for-age 85th–94.9th percentile) and obesity (BMI-for-age ≥95th percentile) in youth with DS (age 2–18 years) is high and higher than rates observed in typically developing youth (3–7). For example, a 2016 literature review which included 45 papers published between 1988 and 2015 reported the combined prevalence of overweight and obesity in children and adolescents with DS ranged from 23% to 70% (6) while a 2021 report found the prevalence of overweight and obesity was 49% in a sample of 122 youth with DS living in the United States (4) compared with 39% in youth in the general population (8). Overweight and obesity in youth with DS increases the probability overweight and obesity in adulthood and obesity associated conditions including sleep apnea, type 2 diabetes, Alzheimer's disease, and increased mortality (9, 10). For example, a recent report demonstrated that overweight and obesity were associated with a 3-fold increase in risk for sleep apnea and a 2-fold increase in risk for type 2 diabetes in adults with DS (4).

The etiology of obesity in youth with DS is unclear (11); however, currently available evidence suggests that a combination of physiologic factors such as increased leptin, decreased resting energy expenditure, chronotropic incompetence and hypotonia, lifestyle factors including consumption of a high calorie diet and low levels of physical activity, and comorbidities associated with DS, including hypothyroidism and congenital heart defects, likely play an important role (6). Obesity typically develops in children with DS around 2 years of age with their BMI percentile remaining stable until puberty (∼ age 12) when significant increases in BMI percentile are frequently observed (3, 7, 12). The increases in BMI percentile are likely associated with decreased parental control over both dietary intake and physical activity resulting in increased consumption of high calorie unhealthy foods (12) and low levels of physical activity observed in adolescents with DS (13). These observations suggest that evidence-based treatments, such as multi-component weight management interventions which include recommendations for decreased energy intake, increased physical activity and education/behavioral counseling, are warranted for adolescents with IDD and overweight or obesity. Physiological characteristics associated with DS including hypotonia, hypothyroidism, decreased resting energy expenditure, increased leptin, chronotropic incompetence and altered gait may impact the effectiveness of weight management interventions in individuals with DS; however, data regarding the impact of weight management interventions in individuals with DS is extremely limited. Although several previous trials have demonstrated the effectiveness of multi-component interventions for weight management in samples of children, adolescents, and young adults with a variety of IDs including DS, results have not been reported by specific ID diagnosis (14–19). Data relative to the effectiveness of multi-component weight management interventions specifically for individuals with DS is limited to one small sample trial which compared weight change across 6 and 12 months in adolescents and young adults with DS (age 13–26 years) randomized to a 6 month nutrition and physical activity education intervention (*n* = 10) or a nutrition and physical activity education plus parent-supported behavioral intervention (*n* = 11), followed by a 6-month no contact follow-up (20). Weight change across both 6 and 12 months was minimal; however, significantly greater weight change at 6 and 12 months was observed in the parent supported (6 mos. = −3.4%; 12 mos. = −2.4%) compared with the non-parent supported arm (6 mos. = + 0.6%; 12 mos. = + 2.2%). We are unaware of any publications which have compared the response to weight management interventions between adolescents and young adults with DS with adolescents and young adults with other types of ID.

Our group recently completed an 18-month weight management trial (6 mos. weight loss, 12 mos. weight maintenance) designed to compare diet [conventional meal plan diet (CD) vs. enhanced Stop Light diet(eSLD)] and delivery strategy [individual face-to-face home visit (FTF) vs. individual remote *via* FaceTime^TM^ (RD)] in 110 adolescents and young adults with ID randomized to one of three intervention arms: FTF/CD, RD/CD, RD/eSLD. A detailed description of the rationale, design, and methods for this trial (21) and the results for our primary outcomes, weight change at 6 and 18 months, have been published previously (22, 23). Weight loss at 6 months was clinically relevant and significantly greater in the eSLD compared with the CD arms when both interventions were delivered remotely: RD/eSLD (−6.4%) vs. RD/CD (−2.4%, *p *= .01). However, 6-month weight loss in the CD arms was minimal and did not differ by delivery strategy: FTF/CD (−0.2%) vs. RD/CD (−2.4%, *p *= 0.20). Weight change across 12 months differed significantly by diet (RD/eSLD: −7.0% vs. RD/CD: −1.1%, *p *= .002) but not by delivery strategy (FTF/CD: +1.1% vs. RD/CD: −1.1%, *p *= 0.21). Weight change across 18 months was minimal in all intervention arms and did not differ by diet (RD/eSLD: −2.6% vs. RD/CD: −0.5%; *p *= 0.28) or delivery strategy (FTF/CD: +1.6% vs. RD/CD: −0.5%; *p *= 0.47). The secondary analysis reported herein compares weight change across 6,12, and 18 months between participants with DS (*n* = 53) and participants with other types of ID (*n* = 57) randomized to the FTF/CD, RD/CD, and RD/eSLD arms.

## Methods

### Participant eligibility

Participants satisfying the following criteria were eligible for this trial: *Inclusion*: Age 13–21 years with mild to moderate ID (IQ 40–74), as verified by a primary care physician, body mass index (BMI) ≥85th percentile on CDC growth charts (age ≤19 years) or ≥25 kg/m^2^ (age >19 years), or waist circumference to height ratio > 0.5 which indicates excess central adiposity in children and adolescents (24, 25) and is commonly observed in youth with DS (26), sufficient functional ability to understand directions, communicate through spoken language, living at home with a parent or guardian, and internet access in the home. *Exclusion*: Type 1 diabetes, or Type 2 diabetes treated with insulin, Prader-Willi Syndrome, participation in a weight management program involving diet and physical activity in the past 6 months, eating disorders, serious food allergies, consuming special diets, or the inability to participate in moderate to vigorous physical activity. To enhance the generalizability of our results individuals who used medications for prevalent conditions associated with obesity or other medications commonly prescribed for individuals with ID were allowed to participate. Clearance from a primary care physician was required for all participants.

### Recruitment/randomization

Participants were recruited through contact with local community programs serving adolescents with ID and using print and web advertisements in the target area. Participants were randomized to intervention arms after providing signed informed parental consent/adolescent assent and written physician clearance. Randomization was stratified by BMI percentile (<95th percentile vs. ≥ 95th percentile) for participants aged 19 and younger and by BMI (25.0–29.9 kg/m^2^ vs. ≥30 kg/m^2^) for participants 19 and over. For adolescents with DS, BMI percentile was calculated using the standard CDC growth chart rather, rather than the DS specific growth chart (27) since the DS specific growth charts do not appear to provide better classification of weight status or health risk for youth with DS over the age of 10 compared to the standard CDC growth chart (28, 29). This trial, which was approved by the University's Institutional Review Board and registered on clinicaltrials.gov (NCT02561754), was conducted in the University's local metropolitan area from November 2015 to May 2021.

### Intervention components

#### Diet

##### Energy intake

Energy intake for weight loss (0–6 mos.) was prescribed at 500–700 kcal/d below total daily energy expenditure estimated using the Dietary Reference Intake (DRI) total energy intake equation for overweight boys/girls (30). Recommended energy intake for weight maintenance (7–18 mos.) was estimated using the DRI equation based on participant weight at 6 months with consideration for adolescent growth and development and adjusted as required based on observed changes in weight across the weight maintenance intervention.

##### Enhanced stop light diet (eSLD)

Participants randomized to the eSLD arm were asked to follow the Stop Light Diet (SLD) (31), which categorizes foods by energy content: green (low energy, consume freely), yellow (moderate energy, consume in moderation), and red (high energy, consume sparingly). The SLD was enhanced by encouraging the consumption of high volume, low energy portion-controlled entrées and shakes (HMR Weight Management Services Corp, Boston, MA) and fruits and vegetables. Participants were encouraged to consume a minimum of 2 entrées (200–270 kcal each), 2 shakes (∼100 kcal each), and 5 one-cup servings of fruits and vegetables each day, as well as lower energy foods (green/yellow) from a chart with pictures of foods that were color-coded based on the SLD system.

##### Conventional meal plan diet (CD)

Participants randomized to the CD arms were asked to consume a nutritionally balanced, reduced energy diet which followed the recommendations found on the USDA website ChooseMyPlate.gov (32) and the Dietary Guidelines for Americans (33). Participants were provided with examples of meal plans consisting of suggested servings of grains, proteins, fruits and vegetables, dairy, and fats based on their energy needs and were counseled on appropriate portion sizes required to achieve the prescribed level of energy reduction. During weight maintenance, participants were asked to continue using a CD as recommended during weight loss; however, suggested servings of grains, proteins, fruits and vegetables, dairy, and fats were recalculated based on their energy needs for weight maintenance.

#### Physical activity

Participants in each intervention arm were asked to reach a target of 60 min./day of moderate-to-vigorous intensity physical activity at least 5 days/wk. (total 300 min/wk.) as recommended by the United States Department of Health and Human Services (34). The recommendation progressed from 15 min/day-3 days/wk. at week one (or current activity level if higher) to 60 min/day-5 days/wk. at week 12 and remained at that level through 18 months.

#### Education/behavioral counseling

##### Health educators

Participants were assigned to an individual health educator for the duration of the study. Health educators were registered dietitians, occupational therapists, and individuals with a degree in exercise Science, kinesiology, psychology, or applied behavior analysis. All health educators receive IDD specific training by two dietitians who specialize in working with individuals with IDD. Additionally, they received weight management specific training by shadowing a comprehensive weight management clinic with physicians, nurse practitioners, physician assistants, and dietitians certified in obesity and weight management for 3–6 months. Health educators were randomly assigned to participants in each of the 3 intervention arms to diminish the potential for health educator bias. All health educator/participant sessions were recorded, and intervention fidelity was assessed by comparing recordings with a check list of content to be delivered. On average, behavioral education sessions delivered 96% of the scheduled content. Eighty percent or more of scheduled content was delivered in all behavioral sessions.

##### Education sessions

Participants and parents in all intervention arms were asked to attend ∼30–45 min. sessions with a health educator twice each month for the first 12 months, and monthly during months 13–18. All participants received an iPad® (Apple Inc, Cupertino, CA), provided by the trial. The RD arms were delivered using FaceTime^TM^ on an iPad® while the FTF arm was delivered during a home visit. Behavioral session content and duration were identical in all 3 intervention arms and included strategies to improve weight loss, e.g., social support, self-monitoring, planning, environmental control, self-efficacy, etc. In addition to the lesson, health educators reviewed self-monitoring data for diet, physical activity, and weight in order to answer questions, problem-solve, and provide support). COVID-19 restrictions prohibited FTF contacts with participants between March and June 2020. Therefore, during this period all sessions with participants in the FTF arm were conducted by telephone. Participants who were uncomfortable with attending FTF meetings following the lifting of the COVID-19 restrictions were allowed to continue with telephone meetings from July 2020 through the completion of the trial (May 2021).

#### Self-monitoring

##### RD arms

Participants, with the help of a parent (if needed), were asked to record all food and beverages consumed on the iPad® using the Lose It! app (Fitnow, Boston MA). Self-monitoring of physical activity was completed using a Fitbit® Charge HR wireless activity tracker (Google, LLC, Mountain View, CA) worn on the wrist. To provide feedback regarding weight change, participants in the RD arms were weighed during the FaceTime^TM^ education/behavioral counseling sessions using a calibrated wireless digital scale (Model: WS-30, Withings Inc. Cambridge, MA). Self-monitoring data was accessible to health educators to inform participant counseling during behavioral sessions.

##### FTF arm

Participants, with the help of a parent (if needed), were asked to record daily number of servings of each food group consumed, minutes of daily physical activity, and the number of steps each day assessed by pedometer provided by the trial (Omron HJ-320, Lake Forest, IL) using a hard copy sheets which were developed for individuals with ID (21, 35) and contained pictorial representations of each food category for assistance. Body weight was monitored using a calibrated digital scale (Model #PS6600, Belfour, Saukville, WI) during each behavioral session. FTF participants who completed behavioral sessions by telephone, i.e., COVID protocol, verbally provided self-monitoring data to the health educator; however, body weight, typically obtained during FTF sessions, was unavailable for sessions conducted by telephone. Self-monitoring records were reviewed with participants during each behavioral session to provide feedback and counseling.

### Outcome assessments

#### Demographics/ID diagnosis

Parents completed a brief survey to obtain participant age, race/ethnicity, sex and ID diagnosis. The parent reported ID diagnosis was verified by the participant's primary care physician who also provided medical clearance for participation in the intervention.

#### Anthropometrics

Weight, height, and waist circumference were assessed during FTF home visits at baseline, 6, 12, and 18 months by trained staff blinded to the intervention arm. Weight was measured in duplicate to the nearest 0.1 kg using a calibrated digital scale (Model #PS6600, Belfour, Saukville, WI) with participants wearing shorts and a t-shirt. Standing height was measured in duplicate with a portable stadiometer (Model #IP0955, Invicta Plastics Limited, Leicester, UK). BMI was calculated as weight in kilograms divided by height in meters squared (kg/m^2^) and BMI- z score was calculated using the Center for Disease Control's (CDCs) growth charts (36).

#### Process outcomes

The percentage of behavioral sessions attended, and the percentage of days participants provided self-monitoring data for diet and physical activity across the 18-month intervention were calculated from health educator records.

### Analysis

This is an unpowered post-hoc analysis to compare weight loss and intervention compliance between adolescents with and without DS. Sample characteristics and outcomes were summarized using means and standard deviations for continuous variables and frequencies and percentages for categorical variables. Separate two sample *t*-tests were used to compare the changes in body weight and BMI between adolescents with DS (*n* = 53) and those with other ID (*n* = 57, 42 Autism, 15 other ID) randomized to the FTF/CD, RD/CD, and RD/eSLD arms across 6,12, and 18 months. Differences between adolescents with DS and other ID for changes in BMI-z score were not analyzed as BMI-z score has been shown to be a poor indicator of change in weight status in children and adolescents (37). Between group differences in session attendance and self-monitoring of both diet and physical activity in each intervention group across 18 months were also evaluated using two-sample *t*-tests. All analyses were conducted using SAS 9.4 (Cary, NC).

## Results

### Participants

**Figure 1:**
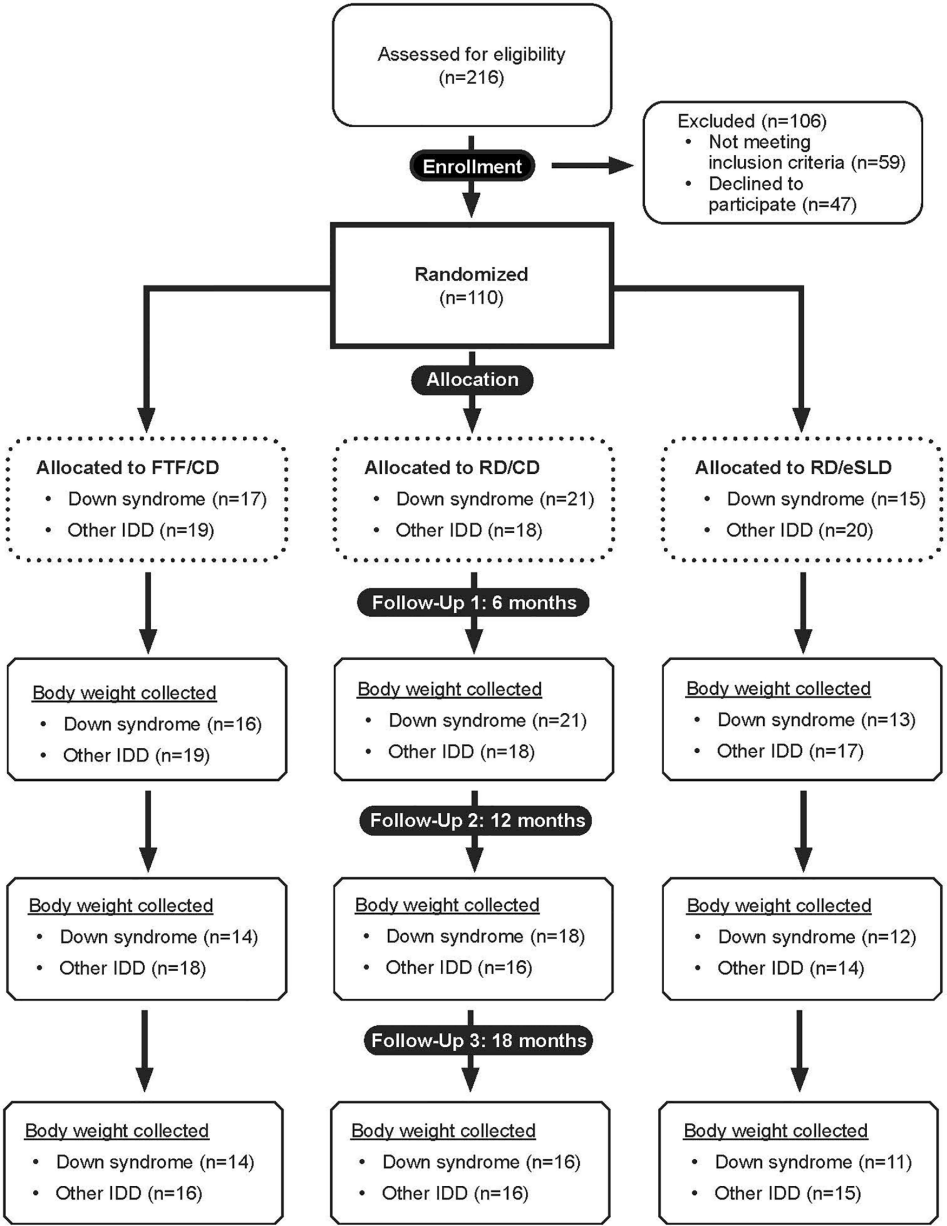
Consort diagram.

Adolescents with ID (*n* = 110) were randomized to one of three intervention arms: FTF/CD (*n* = 36, DS = 17, other ID = 19), RD/CD (*n* = 39, DS = 21, other ID = 18) or RD/eSLD (*n* = 35, DS = 15, other ID = 20). Figure 1 illustrates the participant flow in the three intervention arms across the 18-month trial. Body weight at 18 months was obtained from 82%, 76% and 73% of participants with DS and 84%, 83% and 75% of participants with other ID randomized to the FTF/CD, RD/CD, and RD/eSLD arms, respectively. Baseline characteristics of participants with DS or other ID by intervention arm are presented in Table 1. Participants were∼16 years of age, 52% female, 88% white with a BMI of∼32 kg/m^2^. Baseline weight was significantly greater in adolescents with other ID (92.6 ± 26.5 kg) compared with those with DS (71.1 ± 17.6 kg, *p* = 0.004); however, there were no significant baseline differences between groups in BMI or BMI-z scores.

**Table 1:** Baseline characteristics of adolescents with down syndrome (DS) or other intellectual disabilities (ID) by intervention arm.

	Face-to-face/conventional diet		Remote delivery/conventional diet		Remote delivery/enhanced stop light diet	
	DS (*n* = 17)	Other ID (*n* = 19)	DS (*n* = 21)	Other ID (*n* = 18)	DS (*n* = 15)	Other ID (*n* = 20)
	*M *±* SD*/% (*n*)	*M *±* SD*/% (*n*)	*M *±* SD*/% (*n*)	*M *±* SD*/% (*n*)	*M *±* SD*/% (*n*)	*M *±* SD*/% (*n*)
**Age (years)**	16.2 ± 3.2	16.4 ± 2.2	15.7 ± 1.7	15.6 ± 1.7	17.1 ± 2.5	16.5 ± 2.5
Sex						
Male	47% (8)	63% (12)	29% (6)	50% (9)	47% (7)	50% (10)
Female	53% (9)	37% (7)	71% (15)	50% (9)	53% (8)	50% (10)
**Race**						
White	88% (15)	79% (15)	95% (20)	100% (18)	80% (12)	85% (17)
Black	12% (2)	5% (1)	0% (0)	0% (0)	13% (2)	10% (2)
Two or more Races	0% (0)	16% (3)	4.8% (1)	0% (0)	7% (1)	5% (1)
**Ethnicity**						
Not Hispanic/latino	88% (15)	100% (19)	91% (19)	100% (18)	93% (14)	85% (17)
Hispanic/latino	12% (2)	0% (0)	9% (2)	0% (0)	7% (1)	15% (3)
**Weight (kg)**	72.9 ± 24.2	105.0 ± 29.7	67.2 ± 13.8	84.2 ± 17.8	72.1 ± 15.9	89.4 ± 31.5
**BMI (kg/m^2^)**	31.9 ± 10.1	36.2 ± 7.0	30.7 ± 5.6	31.2 ± 7.5	30.1 ± 6.3	32.3 ± 8.9
**BMI z-score** ^a^	1.86 ± 0.57	2.24 ± 0.56	1.78 ± 0.42	1.87 ± 0.63	1.86 ± 0.45	1.98 ± 0.56

^a^ Calculated for participants ≤ 19 years of age (FTF/CD = 30, RD/CD = 38, RD/eSLD = 27).

**Figure 2:**
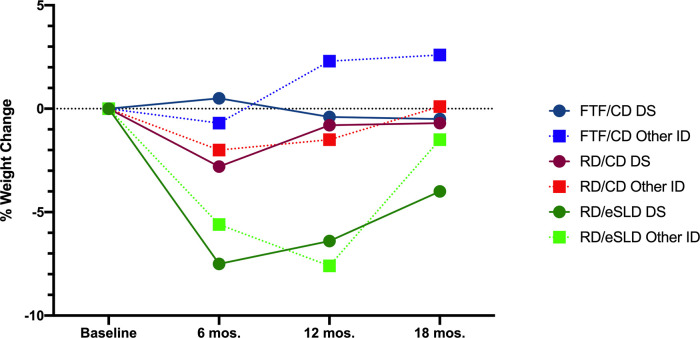
Percent weight change in adolescents with down syndrome (DS) or other intellectual disability (ID) across 18-months by intervention arm.

**Figure 3:**
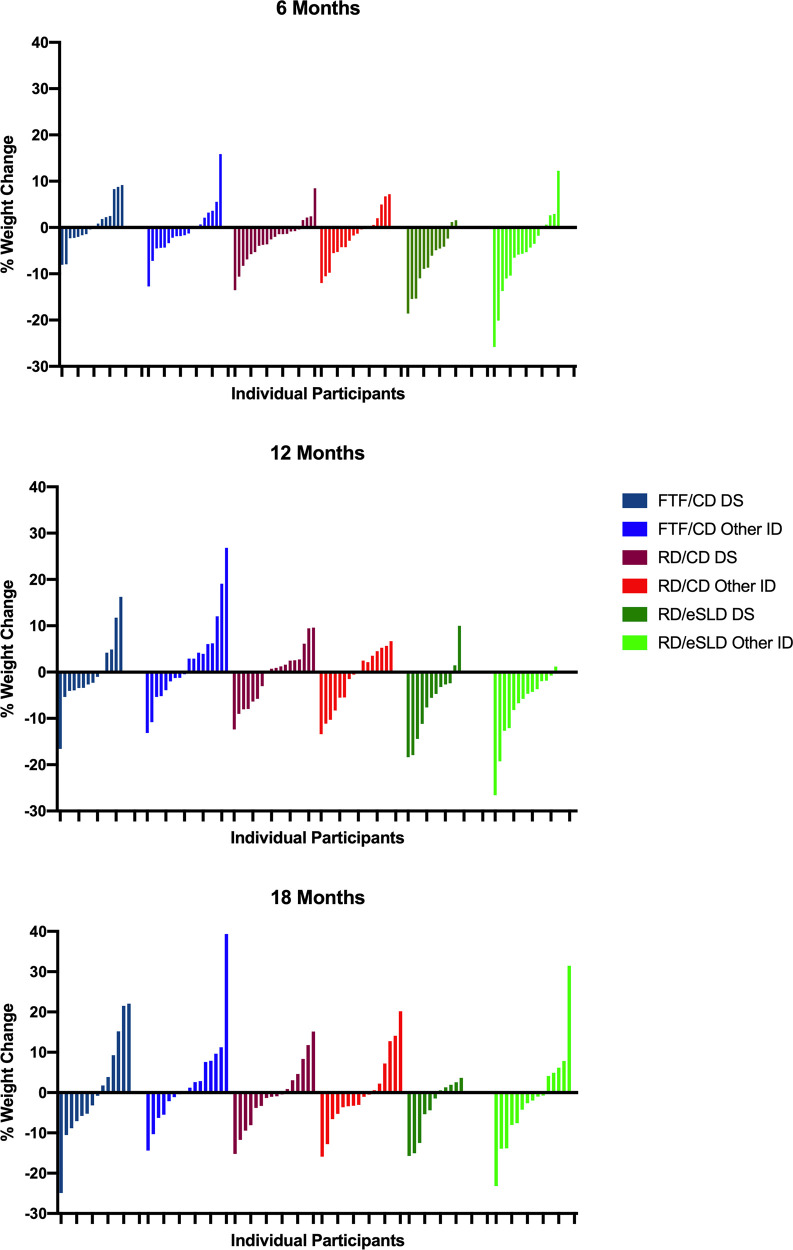
Individual percent weight change at 6, 12 and 18 months in adolescents with down syndrome (DS) or other intellectual disability (ID) by intervention arm.

**Comparison of weight change between adolescents with DS or other ID (**Table 2, Figure 2**)**. We observed no significant differences in change in body weight or BMI across 6, 12 or 18 months between adolescents with DS or those with other ID in any of the 3 intervention arms (all *p* > 0.05). Six-month weight change was 0.2 ± 3.4 kg (0.5%), −1.8 ± 3.1 kg (−2.8%), and −5.1 ± 4.2 kg (−7.5%) in adolescents with DS and −0.7 ± 6.1 kg (−0.7%), −1.8 ± 4.9 kg (−2.0%) and −4.9 ± 7.1 kg (−5.6%) in adolescents with other ID randomized to the FTF/CD, RD/CD, and RD/eSLD arms, respectively. Weight change across 12 months was −0.6 ± 5.5 kg (−0.4%), −0.5 ± 4.1 kg (−2.0%), and −4.0 ± 6.1 kg (−6.4%) in adolescents with DS and −2.8 ± 9.8 kg (2.3%), −1.0 ± 5.4 kg (−1.5%) and −6.4 ± 6.6 kg (−7.6%) in adolescents with other ID randomized to the FTF/CD, RD/CD and RD/eSLD arms, respectively. Weight change across 18 months was −0.2 ± 8.8 kg (−0.5%), −0.3 ± 5.3 kg (−0.7%), and −2.6 ± 5.0 kg (−4.0%) in adolescents with DS and +2.8 ± 10.6 kg (+2.6%), +0.4 ± 8 kg (+0.1%), and −1.8 ± 8.9 kg (−1.5%) in adolescents with other ID randomized to the FTF/CD, RD/CD and RD/eSLD arms, respectively. Figure 3 demonstrates a high degree of individual variability in weight change across 6, 12, and 18 months that is similar in both participants with DS and other ID across the three intervention arms.

**Table 2:** Change in weight and BMI across 6, 12 and 18 months in adolescents down syndrome (DS) and other intellectual disability (ID) by intervention arm.

	Face-to-face/conventional diet					Remote delivery/conventional diet					Remote delivery/enhanced stop light diet				
	DS	Other ID			*p-Value*	DS		Other ID		*p-Value*	DS		Other ID		*p-Value*
	*n*	*M (SD)*	*n*	*M (SD)*	* *	*n*	*M (SD)*	*n*	*M (SD)*		*n*	*M (SD)*	*n*	*M (SD)*	
Across 6-months (0–6 mos.)															
Δ Weight (kg)	16	0.2 (3.4)	19	−0.7 (6.1)	0.63	21	−1.8 (3.1)	18	−1.8 (4.9)	0.99	13	−5.1 (4.2)	17	−4.9 (7.1)	0.93
Δ Weight (%)	16	0.5 (5.1)	19	−0.7 (5.8)	0.52	21	−2.8 (4.8)	18	−2.0 (5.5)	0.66	13	−7.5 (6.3)	17	−5.6 (9.0)	0.55
Δ BMI (kg/m^2^)	16	−0.2 (1.5)	18	−0.5 (1.8)	0.58	18	−1.0 (1.3)	18	−0.8 (2.0)	0.77	12	−2.3 (2.2)	17	−2.1 (2.7)	0.81
Across 12-months (0–12 mos.)															
Δ Weight (kg)	14	−0.6 (5.5)	18	2.8 (9.8)	0.26	18	−0.5 (4.1)	16	−1.0 (5.4)	0.77	12	−4.0 (6.1)	14	−6.4 (6.6)	0.36
Δ Weight (%)	14	−0.4 (8.0)	18	2.3 (9.9)	0.41	18	−0.8 (6.4)	16	−1.5 (6.7)	0.74	12	−6.4 (8.2)	14	−7.6 (7.7)	0.68
Δ BMI (kg/m^2^)	14	−0.8 (2.3)	15	−0.1 (2.6)	0.46	18	−0.5 (2.0)	12	−1.1 (2.5)	0.49	10	−1.9 (3.0)	12	−2.6 (2.7)	0.61
Across 18-months (0–18 mos.)															
Δ Weight (kg)	14	−0.2 (8.8)	16	2.8 (10.6)	0.42	16	−0.3 (5.3)	16	0.4 (8.0)	0.76	11	−2.6 (5.0)	15	−1.8 (8.9)	0.79
Δ Weight (%)	14	−0.5 (13.0)	16	2.6 (12.1)	0.65	16	−0.7 (8.2)	16	0.1 (9.5)	0.80	11	−4.0 (7.2)	15	−1.5 (12.5)	0.55
Δ BMI (kg/m^2^)	10	−0.4 (4.2)	16	0.1 (2.6)	0.74	14	−0.4 (2.3)	16	−0.6 (2.8)	0.87	10	−1.5 (2.5)	15	−1.2 (2.9)	0.82

**Adherence to intervention components** (Table 3**)**. Adherence with intervention components was general high and ranged from 78% to 88% for attendance at education/behavioral counseling sessions, 65% to 85% for self-monitoring of diet, and 68% to 81% for self-monitoring of physical activity. There were no statistically significant differences between participants with DS and those with other ID for these adherence measures in any of the three intervention arms (all *p *> 0.05).

**Table 3:** Adherence to intervention components in adolescents with down syndrome (DS) or other intellectual disability (ID) across 18-months by intervention arm.

	Face-to-face/conventional diet			Remote delivery/conventional diet			Remote delivery/enhanced stop light diet		
	DS	Other ID	*p-Value*	DS	Other ID	*p-Value*	DS	Other ID	*p-Value*
	*M (SD)*	*M (SD)*	* *	*M (SD)*	*M (SD)*		*M (SD)*	*M (SD)*	
Behavioral session attendance	88% (11%)	83% (11%)	0.45	78% (23%)	82% (14%)	0.50	87% (15%)	83% (18%)	0.46
Dietary self-monitoring	85% (25%)	69% (35%)	0.22	65% (32%)	75% (26%)	0.31	80% (27%)	69% (35%)	0.37
Physical activity self-monitoring	81% (25%)	73% (32%)	0.47	73% (28%)	68% (25%)	0.59	80% (25%)	73% (32%)	0.50

## Discussion

The results of this analysis indicate that weight change, attendance at educational/behavioral counseling sessions and compliance with self-monitoring of diet and physical activity in response to an 18-month weight management intervention did not differ significantly between adolescents/young adults with DS and adolescents/young adults with other types of ID. Similar results were observed across two energy reduced diets (CD vs. eSLD) and two strategies for the delivery of educational/behavioral counseling (FTF vs. FaceTime^TM^). Our results suggest weight loss across 6 (−7.5%), 12 (−6.4%) and 18 months (−4.0%) of a magnitude potentially associated with clinical benefits (e.g. lower blood pressure, LDL, fasting glucose) (38) can be achieved in both adolescents with DS and other types of ID who complete a multi-component weight management intervention using an eSLD with remotely delivered education/behavioral counseling (RD/eSLD). Weight loss was achieved in adolescents with DS in spite of the presence of potential obesogenic physiologic characteristics associated with DS including hypotonia, hypothyroidism, decreased resting energy expenditure, increased leptin, and chronotropic incompetence. However, additional trials will be required to evaluate strategies to minimize weight regain after 12 months that was observed in both adolescents with DS and other types of ID in the RD/eSLD arm. In contrast to our results using the eSLD, weight change across 6, 12 and 18 months was minimal (<3%) in both adolescents with DS and other types of ID using a CD delivered either remotely or FTF.

We are unaware of previous trials which have compared weight change between adolescents with DS and those with other types of ID in response to a multi-component weight management intervention. The minimal weight loss across 6 and 12 months observed in adolescents with DS in the current trial using a CD delivered both FTF and remotely is consistent with the results reported by Curtin et al. (20) who observed minimal 6 and 12 month weight loss in 21 adolescents and young adults randomized to a CD with (6 mos. = −3.4%; 12 mos. = −2.4%) and without parental support (6 mos. = + 0.6%; 12 mos. = +2.2%). Our observation of similar weight change in adolescents/young adults with DS and those with other types of ID are in agreement with results from a previously published secondary analysis of data (39) from an 18-month multi-component weight management intervention (6 mos. weight loss, 12 mos. maintenance) in adults with ID (age ∼37 years., BMI ∼37 kg/m^2^) completed by our group (40, 41). For this analysis we estimated a propensity score for the probability of having DS or other types of ID for each participant using a logistic regression model including the following baseline variables as covariates: age, sex, race/ethnicity, BMI, and original study randomization group (eSLD or CD). The analytic cohort included 124 participants, 21 with DS and 103 with other ID. Successful propensity matches were obtained for 20 of the 21 participants with DS. Results indicated weight loss across 18 months was clinically relevant (≥5%) and did not differ significantly between adults with DS (−5.2%) or other types of ID (−6.8%; *p* = 0.39). Thus, the limited available evidence suggests that adolescents with DS or other types of ID respond to multi-component weight management interventions in similar manner.

Strengths of this analysis include the use of data from long-term intervention (18 mos.) that was tailored to the cognitive abilities of adolescents with ID and included both a weight loss (6 mos.) and weight maintenance phase (12 mos.), a similar number of adolescents with DS (*n* = 53,48%) and other types of ID (*n* = 57, 52%), and a high rate of participant retention which ranged from 94% at 6 months to 80% at 18 months. This paper describes results from an unpowered secondary analysis from a randomized trial in a sample of 110 adolescents (*n* = 53 DS, *n* = 57 other ID) that was not specifically designed to evaluate differences in weight change between adolescents with DS and other types of ID. Thus, our sample size for comparisons of weight change between adolescents with DS and other types of ID was small averaging 15 participants with DS and 17 participants with other types of ID within each of the three intervention arms at 6, 12, and 18 months which represents a potential limitation. Additionally, our results are based on a sample of adolescents with mild-to-moderate ID and overweight/obesity living at home with a parent, who volunteered to participate in a weight management trial. Thus, these results may not be generalizable to adolescents with more severe ID, those living in group homes or other living arrangements, or outside of the context of a research trial.

In summary, despite physiologic characteristics associated with DS that may contribute to the development of obesity and hinder the ability to lose weight, the results of this analysis suggest that adolescents with DS can achieve potentially clinically meaningful weight loss across 18 months in response to a multi-component weight management intervention tailored to their cognitive ability using an eSLD with remotely delivered education/behavioral counseling. Additional trials/analyses to confirm these results and to evaluate the influence of factors such as ID severity and living arrangement and to explore strategies to minimize weight regain observed during the final 6 months of the weight maintenance intervention are warranted.

## Data Availability

Deidentified individual participant data (including data dictionaries) will be made available, in addition to study protocols, the statistical analysis plan, and the informed consent form. The data will be made available upon publication to researchers who provide a methodologically sound proposal for use in achieving the goals of the approved proposal. Proposals should be submitted to the corresponding author at lptomey@kumc.edu.
